# Preliminary Assessment of Alkaloid Content in Cocoa (*Theobroma cacao* L.) Hulls for Safe Consumption as a Feed Ingredient

**DOI:** 10.3390/toxins17090441

**Published:** 2025-09-03

**Authors:** Francesca Mercogliano, Corinne Bani, Marco Tretola, Carla Landolfi, Matteo Ottoboni, Federica Cheli, Patrizia Restani, Luciano Pinotti, Chiara Di Lorenzo

**Affiliations:** 1 Department of Pharmacological and Biomolecular Sciences, Università Degli Studi di Milano, Via Balzaretti 9, 20133 Milano, Italy; francesca.mercogliano@unimi.it (F.M.); corinne.bani@unimi.it (C.B.); 2 Institute for Livestock Sciences, Agroscope, La Tioleyre 4, 1725 Posieux, Switzerland; marco.tretola@agroscope.admin.ch; 3 ToxHub Srl, Via Ariberto 20, 20123 Milan, Italy; carla.landolfi@toxhub-consulting.com; 4 Department of Veterinary Medicine and Animal Sciences, Università Degli Studi di Milano, Via Dell’Università 6, 26900 Lodi, Italy; matteo.ottoboni@unimi.it (M.O.); federica.cheli@unimi.it (F.C.); luciano.pinotti@unimi.it (L.P.); 5 Faculty of Pharmacy, Università Degli Studi di Milano, 20133 Milano, Italy; patrizia.restani@unimi.it; 6 Coordinating Research Center (CRC) “Innovation for Well-Being and Environment”, Università Degli Studi di Milano, 20133 Milano, Italy

**Keywords:** cocoa by-products, circular economy, theobromine, caffeine, animal dietary exposure

## Abstract

The European Circular Economy Action Plan outlines a forward-looking strategy that emphasizes waste reduction and the acquisition of high-quality secondary resources. Previous research has shown that cocoa processing by-products contain compounds of interest for various industrial areas, making them an attractive matrix for reuse. However, a gap remains in our understanding of the safety of these by-products intended for feed. In this study, theobromine and caffeine were quantified by High-Performance Liquid Chromatography (HPLC-UV) in cocoa hulls for safety considerations, evaluating theobromine compliance with toxicological and safety levels, and considering their potential application as an ingredient in animal feed. In addition, the identification of phenolic components and associated antioxidant activity was conducted through High-Performance Thin-Layer Chromatography (HPTLC). This preliminary study indicates that theobromine content is a limiting factor for the inclusion of cocoa hulls in animal diets, as it restricts inclusion levels to remain within current regulatory limits. Examples of general estimates of dietary theobromine exposure at inclusion levels based on regulatory limits for dairy cows and veal calves confirmed a low risk for animal health. Furthermore, the detection of antioxidant activity linked to the presence of polyphenols highlights the potential of cocoa hulls as a sustainable food by-product for feed formulation.

## 1. Introduction

The food value chain is responsible for significant resource and environmental pressures, and it is estimated that in Europe, around 20% of the total food produced is lost or wasted [1]. The European Circular Economy Action Plan provides a future-oriented agenda for achieving a cleaner and more competitive Europe. In this context, it is essential to reduce waste and ensure high-quality secondary raw materials [1,2,3,4,5]. The valorization of cocoa (*Theobroma cacao* L.) processing waste has generated great interest from both nutritional and functional points of view; notably, about 80% of processed cocoa fruit is discarded [6], highlighting the substantial volume of generated waste. This extensive production of residual biomass poses a significant environmental issue for countries involved in cocoa cultivation and production [6,7], but also in the EU, where chocolate production occurs and therefore processing waste is produced.

On the other hand, cocoa by-products (e.g., cocoa pod shells, mucilages, and hulls) contain compounds of interest for various economic areas, such as food and the cosmetic and chemical industries [8]. In fact, cocoa bean by-products contain an interesting profile of polyphenols (flavonols, phenolic acids) [9,10], methylxanthines, dietary fibers, and lipids [11]. In the year 2020–2021 (from the 1st of October to the 30th of September), 5245 thousand tonnes of cocoa beans were produced worldwide [7], leading to a substantial amount of waste generated from their processing. From this production, each year, an estimated 700 to 900 thousand tons of cocoa hulls (CHs) are produced worldwide, with approximately 300 thousand tons of waste generated in Europe [11]. CHs, also called cocoa bean shells, are the by-product of the dehulling step in the extraction of cocoa butter; they are removed from the beans and often discarded as waste. Interest has therefore been raised in healthy ingredients that can perform positive functions, such as antioxidant protection and anti-inflammatory action, but also for ingredients that would decrease feed–food competition. There are some studies evaluating alternative uses for food by-products, for example, the production of prebiotic and functional ingredients [12], and the most common applications include uses as biofuel, activated carbon preparation, mulch and fertilizer, and feedstuff for livestock [13]. CHs, being a rich source of carbohydrates, dietary fiber, protein, ash, and polyphenols such as quercetin, epicatechin, and catechin [14,15], are an interesting matrix for reuse, as in the case of animal feed, in line with the circular economy aim of optimizing available resources and reducing food waste [16]. Indeed, modern animal husbandry includes, among its objectives, the study of new dietetic formulations in order to improve animal welfare [17].

According to INRAE-CIRAD-AFZ (French National Institute for Agricultural Research–French Agricultural Research and Cooperation Organization–French Association for Animal Production) feed tables [18], CHs contain approximately 17.8% of crude protein on dry matter (DM), 5.9% of ether extract and 7.8% of starch, leading to a gross energy of 4490 kcal/kg on DM. CHs also contain a high insoluble fiber content, around 46.5%, and minerals including phosphorus and magnesium. This chemical composition makes CHs particularly interesting as ruminant feed ingredients [19]. In addition, the presence of tannins (1.70–25.30 mg/g) [13] contributes both functional benefits and challenges; at appropriate levels, tannins can improve protein utilization by reducing excessive ruminal protein degradation and help control bloat and parasites, while excessive amounts may negatively affect nutrient availability and animal health [20].

While CHs offer potential for reuse in animal feed due to their nutritional value, the presence of naturally occurring methylxanthines, particularly theobromine and caffeine, poses a significant toxicological concern that may limit the use of CHs in animal nutrition. Toxic effects of theobromine have been documented in several species. In an EFSA Scientific Opinion [21], the Contaminants in the Food Chain Panel reported toxicological studies where theobromine showed toxicity in rodents, with target organs the testes and thymus; the study allowed for the definition of a No Observed Adverse Effect Level (NOAEL) for testicular toxicity of 150 mg/kg b.w. in rats. Theobromine also showed adverse effects in skeletal development of rabbit offspring, leading to a NOAEL of 21 mg/kg b.w. Adverse effects were also reported in target animals. For example, pigs showed growth retardation, diarrhea, and lethargy [22]. Horses and dogs, which are especially sensitive to theobromine, showed liver and thyroid damage and fatal intoxications, respectively. The mutagenic and clastogenic effects of theobromine were generally reported as equivocal; no long-term carcinogenicity studies are available, and no ADI (acceptable daily intake) has been established [21]. So overall, the EFSA Panel stated that there is a general lack of data regarding the feed theobromine levels able to determine a negative effect on animals and underlined the need for further information on the use of various feed materials containing cocoa.

The European Union (EU) has recognized these risks, and Directive 2002/32/EC of the European Parliament and of the Council lists several compounds that are considered undesirable, including theobromine, in animal feeds and prescribes their ML for different feed commodities. The current theobromine EU ML for complete feed with a moisture content of 12% is 300 mg/kg, apart from that for pigs, for which the maximum content is set at 200 mg/kg, and for dogs, rabbits, horses, and fur animals, for which it is set at 50 mg/kg of feed, as last amended in 2019.

Differently for theobromine, the Directive (2002/32/EC) does not regulate caffeine, which also demonstrates toxicological relevance. In a 90-day toxicity study in rats and mice, caffeine led to a slight body weight decrease with no toxicity signs [23]. Numerous genotoxicity data are available showing no evidence of mutagenic effects for this molecule; reproductive effects were observed alongside general toxicity in parental mice with a NOAEL of 22 mg/kg b.w. for F0 parental and F1 offspring, and 88 mg/kg b.w. for F1 parental and F2 offspring [23].

Despite these known risks, various *Theobroma cacao* L. by-products (i.e., cocoa husks, hulls, and bean meal) are on the Catalogue of Feed Materials (Reg. EU 2022/1104) [24] and Cocoa absolute CoE 452 is authorized as a feed additive in the European Union (EU) (Council Directive 70/524/EEC) [25].

In response to this dual context of nutritional potential and toxicological risk, the present study aims to contribute to the development of evidence-based strategies for the safe and sustainable incorporation of cocoa by-products into animal feed formulations. This study aims to validate a High-Performance Liquid Chromatography method with ultraviolet detection (HPLC-UV) for the quantification of theobromine and caffeine in cocoa hulls (CHs) and to evaluate their compliance with EU safety limits for animal feed. Additionally, this study investigates the presence of polyphenols and antioxidant activity to support the valorization of CHs as a sustainable ingredient within a circular economy framework.

## 2. Results and Discussion

### 2.1. Development and Validation of the High-Performance Liquid Chromatography (HPLC) Method

**Figure 1:**
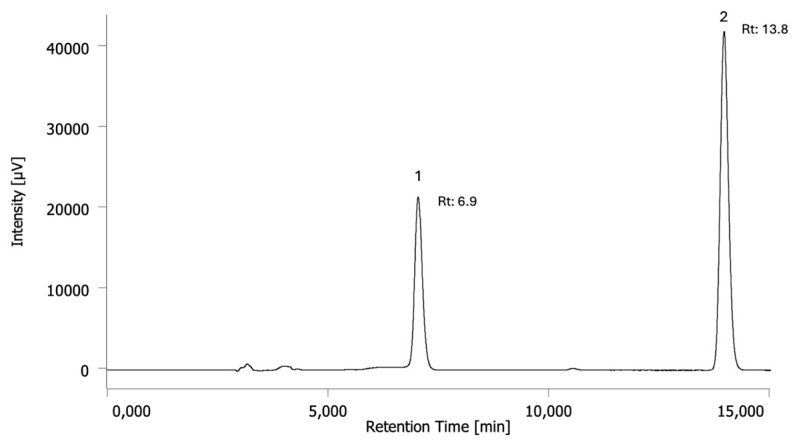
Chromatographic profiles of a mix of the standards at a concentration of 10 µg/mL and their retention times (Rt) (min). Peak 1: theobromine; peak 2: caffeine.

The HPLC-UV method was developed to quantify alkaloids in CHs. The method was validated, according to FDA recommendations [26], to quantify theobromine and caffeine. Validation parameters are illustrated in Table 1, and chromatographic patterns are reported in Figure 1.

A correlation coefficient (R^2^) greater than 0.98 (Table 1) was obtained for both standards and demonstrated a good linearity for the selected range of concentrations. The sensitivity of the method, defined as the lowest concentration of analyte that can be detected (LOD) or quantified (LOQ), is illustrated in Table 1. The recovery, which includes efficiency and reproducibility of the extraction, was confirmed within the range of 80–115% as required by the validation test [26]. The stability in the short term (after 24 h) and long term (after 20 days) showed a percentage of variation within ±10% and ±15%, respectively. Precision, assessed as intraday and interday variation, showed a coefficient of variation (CV%) of less than 15%, as required [26].

### 2.2. Measurement of Theobromine and Caffeine by HPLC and Safety Considerations

**Figure 2:**
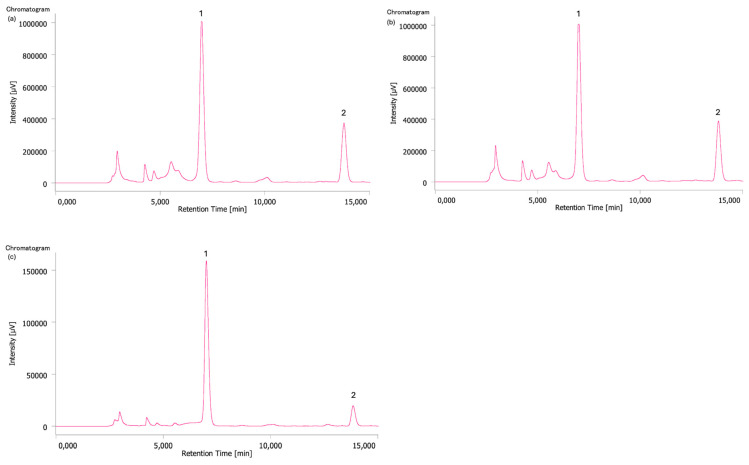
Chromatograms of samples S1 (**a**), concentrated 3:1; S2 (**b**), concentrated 3:1; and S3 (**c**), diluted 1:10. Peak 1: theobromine (Rt: 6.9); peak 2: caffeine (Rt: 13.8).

In all samples, theobromine was found at higher concentrations than caffeine. Sample 3 (S3), which is in pellet form, contained a higher concentration of analyzed analytes when compared to all samples in flake form (S1 and S2). This suggests that the pellet form may preserve or concentrate these compounds more effectively than the flake form [27]. These results agree with the results obtained by the HPTLC method (Section 2.3), where the comparison between the samples showed a higher presence of analytes in sample S3 than in samples S2 and S1. Figure 2 shows the HPLC profile of S1 (a), S2 (b), and S3 (c).

Peaks 1 and 2 (Figure 2) were identified as theobromine and caffeine, respectively; their quantification in µg/g is reported in Table 2.

CHs contain CNS-stimulating alkaloids, primarily caffeine and theobromine, and constituents that could be classified as toxic or anti-nutritious, such as biogenic amines, tannin and trypsin inhibitor, even if poorly investigated [21]. It is therefore essential to propose a suitable formulation in case of the inclusion of cocoa or its by-products into animal feed.

The quantification by HPLC-UV performed in this study (Table 2) showed a theobromine concentration of approximately 0.400 g/100 g for samples in flakes form and 0.550 g/100 g for pellet form. In particular, theobromine concentration ranged between 4036.65 ± 80.53 µg/g for S2 and 5463.44 ± 109.84 µg/g in S3 (Table 2). These results are consistent with data previously published, where the theobromine content in CHs was in a range of 0.39–1.83 g/100 g [13]. To remain within the EU ML set for theobromine in complete feed (300 mg/kg, with exceptions for pigs, dogs, rabbits, horses, and fur animals, for which it is set at lower levels) [28], the CHs analyzed in this study could be used in a ruminant diet with an inclusion rate of a maximum of 7.14% for S1, 7.42% for S2, and 5.49% for S3. These values should be adjusted if other sources of theobromine (e.g., tea leaves, herbal feed supplements) are present in the proposed diet. To provide examples of potential theobromine exposure from CHs in ruminant diets, calculations are provided for dairy cows and veal calves. Using the calculated inclusion rates of CHs and assuming default values for live weight of 650 kg for dairy cows and 100 kg for veal calves [29], and default values for feed intake of 20.0 kg/day and 1.89 kg/day, respectively [29], the animal daily theobromine intake can be estimated at 9.23 mg/kg b.w. for dairy cow and 5.67 mg/kg b.w. for veal calves. These estimated exposure levels, based on inclusion rates aligned with the current regulatory limit of 300 mg/kg, fall below thresholds associated with observed effects in the literature. Specifically, previous studies showed no health effects in cows at around 23 mg/kg b.w. [30], with milk yield and composition affected only at higher intakes of 14 to 45 mg/kg b.w. [31]. Calves exhibited toxicity only at higher doses of 45 to 90 mg/kg b.w. [32]. These findings indicate that the current regulatory limit and calculated CH inclusion rates ensure low risk exposure to theobromine in both dairy cows and veal calves.

While this study provides valuable insights, it is important to recognize that the analysis was limited to three CHs batches. As such, the findings may not comprehensively reflect the full range of compositional variability that could be present across different sources or production lots. The theobromine content of the raw materials can vary significantly depending on origin, processing, and storage conditions. Furthermore, the effects of feed processing techniques, such as grinding, pelletization, or extrusion, on the stability and bioavailability of theobromine may also influence the actual risk of toxicity in practical feeding scenarios [33]. More research involving a larger sample size, diverse sources of CHs, and different production processes is needed to validate these findings and improve the robustness of animal dietary recommendations.

Previous studies on the influence of CHs on animals, with different inclusion rates, have demonstrated species-specific outcomes. In poultry, CHs affected performance based on inclusion level and treatment: untreated CHs reduced broiler weight and egg production at 4% and 6% inclusion levels [34,35], but hot water-treated CHs could be safely used up to 20% in layers’ diets [36,37]. In rabbits, untreated CHs were tolerated at 100 g/kg, and hot water-treated CHs at 200 g/kg, supporting growth and profitability [38]. In pigs, CHs improved gut microbiota and health markers [37], with 20% being optimal as a maize substitute [39]. In aquaculture, cocoa by-products supported growth in Clarias gariepinus [40] and Nile tilapia [41], though bitterness limited the intake. For ruminants, the inclusion of 40% cocoa bean waste (mixture of CHs, cocoa pulp, and cocoa placenta) as a feed source improved daily weight gain and feed efficiency in cattle [42], while the inclusion of 12% of CHs enhanced milk quality in ewes without affecting yield [43]. Early-lactating dairy cows also benefited from CH inclusion without health or methane production [19]. Goats fed diets with CHs and lignocellulosic materials showed favorable weight gains and feed conversion [44], and similar benefits were observed in dairy goats without affecting milk yield [45]. In vitro digestibility studies further confirmed the nutritional value of CHs, particularly when ensiled with poultry manure, which improved nutrient availability, reduced anti-nutritional factors, and demonstrated high organic matter digestibility and metabolizable energy, making it a viable and eco-friendly feed option for ruminants [46]. While these findings highlight the potential of CHs as a sustainable livestock feed ingredient, especially for ruminants, species-specific sensitivities must be taken into account. Notably, dogs are particularly vulnerable, with reported clinical signs, including cardiovascular, neurological, and gastrointestinal effects, after chocolate ingestion [21]. Due to the documented sensitivity of dogs to theobromine toxicity, feed manufacturers exclude cocoa by-products from feeds for dogs [21].

In response to such concerns, several detoxification strategies have been developed to reduce alkaloid levels in cocoa by-products and enhance their safety for use in animal feed [47]. Various strategies have been explored, especially to reduce the theobromine content: physicochemical methods like 15 min boiling have shown a good balance between theobromine reduction and nutrient retention [36], while alkali treatments also reduce theobromine but at the cost of essential nutrients [48]. Biological treatments offer a more nutrient-preserving alternative. Fungi such as *Aspergillus niger*, *Talaromyces verruculosus*, and *T. marneffei* have demonstrated strong detheobromination potential [49,50]. *T. verruculosus* TvTD was also identified as a suitable bio-tool for cocoa by-product detoxification [51], with additional evidence suggesting it also degrades other methylxanthines like caffeine. However, many regulatory authorities require a thorough evaluation of the safety and efficacy of microbial detoxification products before they can be approved for use [52]. In parallel to detheobromination, supercritical CO_2_ extraction has been used to selectively remove caffeine up to 80% from cocoa products while retaining theobromine, polyphenols, and antioxidant activity [53].

Besides considering safety, toxicological limits, and detoxification strategies, nutritional considerations and the potential variations in caffeine and theobromine content in different batches and sources of these by-products must also be considered during the diet formulation phase to ensure accurate and safe dietary practices. Moreover, it is crucial to acknowledge the challenges associated with variability in CHs sourced from complex supply chains, such as those of cocoa production. The process of acquiring cocoa from multiple suppliers in different countries worldwide creates a complex network for large production chains [54]. Cocoa production companies prioritize consistency in their products over time, which requires suppliers to deliver cocoa with uniform characteristics [55]. This commitment to product uniformity also extends to by-products like CHs, where consistent quality standards are maintained. Nevertheless, conducting quality assurance analyses alongside safety assessments is important to ensure that these standards are consistently met and to prevent the distribution of animal feed products that may contain undesirable substances (e.g., theobromine) with levels exceeding the EU’s ML, as prohibited in Directive 2002/32/EC.

### 2.3. Screening of Other Constituents of Nutritional Interest by High-Performance Thin-Layer Chromatography (HPTLC)

HPTLC analysis allowed for the identification and semi-quantification of the phenolic compounds present in the samples by derivatization with Fast Blue B Salt, while the overall antioxidant activity was appreciated by derivatization with the DPPH solution.

**Figure 3:**
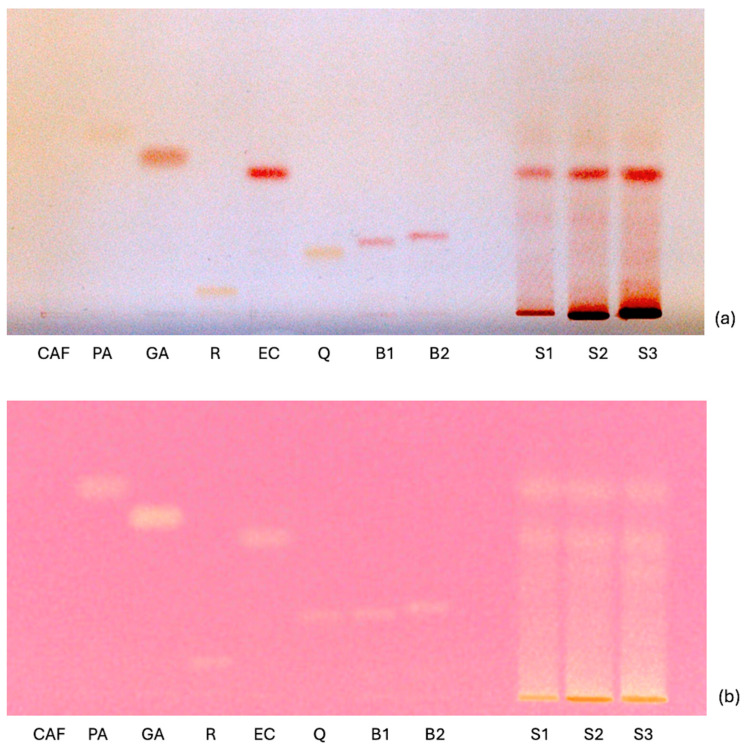
HPTLC plate after derivatization with Fast Blue B Salt (**a**) and DPPH (**b**), detected by visible light. CAF: caffeine; PA: protocatechuic acid; GA: gallic acid; R: rutin; EC: epicatechin; Q: quercetin; B1: procyanidin B1; B2: procyanidin B2; S1: sample 1; S2: sample S2; S3: sample S3.

The HPTLC patterns, detected at visible light, are shown in Figure 3.

The samples, illustrated in Figure 3a, have a significant content of phenolic compounds, which is higher for sample S3 when compared to S2 and S1. The epicatechin band is present in all the samples (Rf = 0.46). Considering Figure 3b, all the standards included in this study, except for caffeine, have antioxidant capacity, as shown by the discoloration of the corresponding bands. All samples (Figure 3b) show a considerable presence of antioxidant molecules and, in particular, of protocatechuic acid (Rf = 0.59) and epicatechin (Rf = 0.45). Epicatechin is a flavanol, a subgroup of flavonoids, present in several foods, among which the main source is cocoa [56]. In 2012, the EFSA Panel on Dietetic Products, Nutrition, and Allergies published a Scientific Opinion on the health claim related to cocoa flavanols, concluding that they help to maintain endothelium-dependent vasodilation, which contributes to normal blood flow [57]. This reinforces the hypothesis that polyphenolic compounds are integral to the antioxidant efficacy of cocoa by-products, thus underscoring their potential benefits. In the literature, the inclusion of polyphenols in feed rations showed different positive effects, such as enhancing the oxidative stability of meat and meat products and reducing the number of additives, like vitamin E and other synthetic antioxidants [58].

## 3. Conclusions

Our study investigated the potential of CHs, a cocoa by-product, as a source of phenolic compounds and antioxidant activity, although the associated alkaloid content must be suitably controlled. An HPLC method for theobromine and caffeine quantification was validated, and the analysis confirmed theobromine as the predominant analyte. S3 contained the highest concentrations of all analyzed compounds compared to S1 and S2. The HPLC quantification of theobromine showed that theobromine is a limiting factor for including CHs in animal diets. The inclusion of CHs analyzed in this study should not exceed 7.14% for S1, 7.42% for S2, and 5.49% for S3 in the ruminant diet to meet the EU’s maximum theobromine limits in complete feed. These inclusion values are specific to the limited sample size of CHs examined in this study and may not reflect the broader variability of theobromine content in CHs. Such variation may also arise from the different geographic origins of the raw materials and the influence of different processing methods. General estimates of dietary theobromine exposure for dairy cows and veal calves were performed, confirming a low risk to animal health when the CH inclusion levels are based on regulatory limits. In addition, the HPTLC analysis confirmed the presence of phenolic compounds and the associated antioxidant capacity in all samples. Further research is needed to support broader dietary recommendations, including CH inclusion rates in animal diets. Adjustments should also be made when other sources of theobromine, such as tea leaves or herbal feed supplements, are present in the diet. Additionally, this study suggests that establishing maximum caffeine limits in feed, as they exist for theobromine, would contribute to ensuring animal safety in response to evolving feed practices, such as the use of food by-products in feed.

## 4. Materials and Methods

### 4.1. Materials

Methanol, ethanol, HPLC-grade water, acetonitrile, acetone, toluene, n-hexane, reagents for analysis, and acids were purchased from VWR International (Fontenay-sous-Bois, France). 1,1-diphenyl-2-picryl-hydrazyl free radical (DPPH), Fast Blu B Salt, caffeine, theobromine, epicatechin, gallic acid, procyanidin B1, procyanidin B2, quercetin, and rutin were purchased from Sigma Aldrich (Merck, Steinheim, Germany).

### 4.2. Samples, Particle Size Determination, and Extraction Method

CHs included in this study were provided by a Swiss former foodstuff processor and consisted of sample 1 (S1) and sample 2 (S2), from different batches of flake-form product, and sample 3 (S3) in pellet form. All samples were maintained at −20 °C upon receipt and until use. The particle size of samples was determined upon arrival by dry sieving analysis for flake samples and wet sieving for pellet particle size, performed according to ISO 2591-1 standard [59], using laboratory sieves ranging from 125 to 4000 μm (Endecotts Ltd., London, UK). Sieving analysis of CHs (Table 3) showed the same particle size distribution in S1 and S2. This was expected since the different samples underwent the same flaking process, while S3 underwent a pelletization process.

To ensure uniformity and accuracy in analytical testing, samples were homogenized.

Each (0.5 g) sample was defatted 4 times with 5 mL of n-hexane by mechanical stirring. Five mL of 80:20 (*v*/*v*) methanol/water solution was added to the defatted samples, homogenized with Ultra-Turrax (IKA T-25; IKA. Staufen, Germany) at 17,000 rpm for 2 min, and centrifuged at 3000× *g* at 4 °C for 14 min (Centrifuge 5810R, Eppendorf, Hamburg, Germany). The extract was then filtered with a 0.45 μm PTFE filter (VWR, Fontenay-sous-Bois, France) and kept at −20 °C until analyzed. To evaluate the reproducibility of the extraction, the process was repeated three times. Each analysis was then performed in triplicate.

### 4.3. High-Performance Thin-Layer Chromatography (HPTLC)

HPTLC is a chromatographic technique that allows for the separation of compounds based on the different affinity of the analytes for the stationary phase (silica gel) and the mobile phase. Through HPTLC analysis, it is possible to carry out qualitative and semi-quantitative analyses regarding the content of phenolic compounds and the antioxidant capacity of the samples [60].

Aliquots of 5 μL of standard solutions (gallic acid, caffeine, epicatechin, procyanidin B1, procyanidin B2, quercetin, and rutin) at the concentration of 200 μg/mL were loaded onto HPTL silica gel plates 60 F254 (dimensions: 10 × 20 cm, manufacturer: Merck, Darmstadt, Germany) by a semi-automatic sample applicator (Linomat 4, CAMAG, Muttenz, Switzerland).

Sample volumes of 10 μL, prepared as described in Section 4.2, were also loaded onto the plates following the procedure published by Colombo and colleagues [61].

After the chromatographic run, where the mobile phase consisted of 10 mL of acetone/toluene/formic acid (in a ratio of 4.5:4.5:1 *v*/*v*/*v*), the plates were exposed to UV light at 254 and 366 nm. Subsequently, they were derivatized with a 0.05% DPPH methanolic solution, kept in the dark for 30 min, and then examined under visible light using VisionCats software v. 1.3.12236.2 (CAMAG, Muttenz, Switzerland). The same operating protocol was also performed for the plate derivatized with Fast Blue B Salt (dianisidine-bis-(diazotised)-zinc double salt).

### 4.4. High-Performance Liquid Chromatography Coupled with Ultraviolet Detection (HPLC-UV)

An HPLC method coupled with an ultraviolet (UV) detector was employed for the quantification of theobromine and caffeine. The gradient elution, set at a flow rate of 1 mL/min, was obtained by mixing the following mobile phases: A, formic acid/water (0.5% *v*/*v*); B, formic acid/acetonitrile (0.5% *v*/*v*). The gradient was set up as follows: 0–30 min: 90–75% A; 30–35 min: 75–0% A; 35–39 min: 0% A isocratic; 39–40 min: 0–90% A; 40–50 min: 90% isocratic A. The UV detector was set at 280 nm, and the column was kept at room temperature (≅25 °C). The method was validated according to the Food and Drug Administration (FDA) Guidelines on Bioanalytical Method Validation [26] by calculating their linearity, sensitivity, recovery, stability, and precision. Linearity was estimated by the correlation coefficient (R^2^). Sensitivity was evaluated by determining the limit of detection (LOD) and quantification (LOQ) at a signal-to-noise ratio of 3 and 10, respectively. The recovery was assessed as extraction efficiency by adding different concentrations of the standard solutions to sample S2. The stability of standards was evaluated in the aliquot extracted and maintained at −20 °C after different storage times (24 h and 20 days). Precision was assessed by calculating the intraday and interday precision expressed as a coefficient of variation (CV%).

Standard stock solutions were prepared at a concentration of 500 µg/mL in methanol/water 80:20 (*v*/*v*). Each standard was suitably diluted to its final concentration range of 10–100 μg/mL in methanol/water 80:20 (*v*/*v*). All solutions were stored at −20 °C until use. Samples were prepared as described in Section 4.2, analyzed in triplicate (*n* = 3) as such, suitably concentrated 3:1 or diluted 1:10, and added with methanol/water 80:20 (*v*/*v*) before the analysis.

The chromatographic separations were carried out on a reversed-phase YMC-Triart C18 column (250 mm, particle size 3.0 µm). The HPLC equipment (Jasco, Tokyo, Japan) consisted of two pumps (model PU-1580), an interface (LC-NETII/ADC), an autosampler (model AS-2059 plus), a degasser (DG-2080-54), a UV detector (model UV-975), and an injection valve (Rheodyne, Cotati, CA, USA) with a 100 mL loop. ChromNAV software v.1.18.03 (Jasco, Tokyo, Japan) was used for data acquisition and processing.

The following formula was used to compute the permissible percentage of samples to be included in feed to comply with the EU ML set for theobromine of 300 mg/kg; exceptions exist for pigs, dogs, rabbits, horses and fur animals, for which lower levels are set (Directive 2002/32/EC) [28]:

% permissible CHs in feed: ML/TC × 100(1)

where ML: EU maximum level for theobromine (mg/kg); TC: theobromine content (mg/kg).

The following formula was used to estimate animal dietary exposure [29]:

Estimated daily animal dietary exposure: (TCF × FI)/b.w.(2)

where TCF: theobromine content in feed (mg/kg); FI: feed intake (kg/day); b.w.: body weight (kg).

### 4.5. Statistical Analysis

Descriptive statistical analyses for calculating the mean values, the standard error of the mean and the correlation coefficient (R^2^) were performed using IBM SPSS Statistics for Macintosh software v. 29.0.2.0 (IBM Corp, Armonk, NY, USA). In addition, data were analyzed with one-way ANOVA when normality and homogeneity of variances were met. Normality was assessed using the Shapiro–Wilk test, while homogeneity of variances was evaluated with Levene’s test. For data that did not meet these assumptions, the Kruskal–Wallis test was used. Significant differences were determined by post hoc Tukey test for ANOVA and Bonferroni for the Kruskal–Wallis test, with significance set at *p* < 0.05.

## Figures and Tables

**Table 1:** HPLC-UV validation parameters for the theobromine (TB) and caffeine (CAF).

	Linearity		Sensitivity				Recovery	Stability		Precision	
	Linear range	R^2^	LOD		LOQ		%	Variation%		Intraday	Interday
	(μg/mL)		(ng/mL)	(ng/g)	(ng/mL)	(ng/g)		24 h	20 days	(CV%)	(CV%)
TB	10–100	0.9820	3	30	10	100	95	1.43	7.74	3.11	6.93
CAF	10–100	0.9973	10	100	40	400	84	2.54	9.53	2.72	6.27

**Table 2:** Content of theobromine (TB) and caffeine (CAF) in the CHs analyzed by HPLC-UV and expressed as mean ± SD (µg/g); *n* = 3. Values with the same letter are not statistically different (*p* > 0.05).

Compound	S1	S2	S3
TB	4199.3 ± 86.97 ^a^	4036.65 ± 80.53 ^a^	5463.44 ± 109.84 ^b^
CAF	349.57 ± 13.19 ^a^	259.17 ± 6.46 ^a^	535.51 ± 16.84 ^b^

**Table 3:** Cumulative particle size (μm) of samples S1, S2 and S3 expressed as mean (%) ± SD; *n* = 3. Values with the different letters are statistically different (*p* < 0.001).

Sieve Openings (µm)	Cumulative Particle Size		
	S1	S2	S3
4000	100	100	100
2000	85.15 ± 0.09 ^a^	85.47 ± 0.08 ^a^	98.89 ± 0.10 ^b^
1000	33.93 ± 0.11 ^a^	33.69 ± 0.30 ^a^	97.31 ± 0.14 ^b^
800	14.69 ± 0.16 ^a^	14.24 ± 0.13 ^a^	95.25 ± 0.04 ^b^
630	11.63 ± 0.26 ^a^	11.64 ± 0.06 ^a^	93.94 ± 0.06 ^b^
400	9.40 ± 0.20 ^a^	9.78 ± 0.01 ^a^	6.40 ± 0.41 ^b^
250	5.72 ± 0.13 ^a^	6.60 ± 0.07 ^a^	3.70 ± 0.33 ^b^
125	3.33 ± 0.10	4.26 ± 0.04	0.00
0	0.61 ± 0.09	0.71 ± 0.03	0.00

## Data Availability

The original contributions presented in this study are included in this article. Further inquiries can be directed to the corresponding author.

## Abbreviations

The following abbreviations are used in this manuscript:

ADI	Acceptable daily intake
b.w.	Body weight
CAF	Caffeine
CH	Cocoa hull
CSN	Central nervous system
CV	Coefficient of variation
DPPH	1,1-diphenyl-2-picryl-hydrazyl free radical
EC	Epicatechin
EU	European Union
EFSA	European Food Safety Authority
FDA	Food and Drug Administration
HBA	p-hydroxybenzoic acid
HPLC	High-Performance Liquid Chromatography
HPTLC	High-Performance Thin-Layer Chromatography
LOD	Limit of detection
LOQ	Limit of quantification
ML	Maximum level
NOAEL	No Observed Adverse Effect Level
*Rf*	Ratio frontis
Rt	Retention time
SD	Standard deviation
TB	Theobromine
TC	Theobromine content
